# Biomimetic Chaotic Sensor for Moderate Static Magnetic Field

**DOI:** 10.3390/s21216964

**Published:** 2021-10-20

**Authors:** Wojciech Korneta, Iacyel Gomes, Rodrigo Picos, Michal Zábovský

**Affiliations:** 1 University Science Park, University of Zilina, Univerzitna 8215/1, SK-01026 Zilina, Slovakia; 2 Department of Industrial Engineering and Construction, Universitat de les Illes Balears, 07122 Palma, Spain; ciel33@yahoo.com (I.G.); rodrigo.picos@uib.es (R.P.); 3 Balearic Islands Health Research Institute (IdISBa), 07120 Palma, Spain

**Keywords:** chaotic sensor, static magnetic field, Chua circuit, biomimetics, spike count rate

## Abstract

The effects of a static magnetic field on systems with chaotic dynamical behavior have attracted little attention so far. Here, Chua’s electronic circuit with an inductor placed in a static uniform magnetic field operating in a chaotic double-scroll regime is studied experimentally. The effect of the magnetic field on the duty cycle factor and the spike count rate, with spikes defined by crossings between the scrolls of the double-scroll attractor, is described. A slow monotonic variation in the duty cycle factor and constant spike count rate is observed for magnetic field intensities up to the threshold, where both these metrics change severely; the dynamic trajectory remains on one scroll and spikes disappear. The dependence of the static magnetic field intensity on Chua’s circuit resistivity at the threshold is given. Two biomimetic magnetic chaotic sensors are proposed: one based on one Chua’s circuit and another that can have various transfer functions and is composed of several independent Chua’s circuits.

## 1. Introduction

Static magnetic fields are used in many technologies for daily use and have an impact on humans, microorganisms, plants and animals [1,2,3]. They can be classified as weak (<1 mT), moderate (1 mT to 1 T) and strong (>1 T) magnetic fields [2]. Moderate-intensity static magnetic fields are produced, e.g., by permanent magnets and electromagnets used in household devices, toys and accessories. The magnetic field is sensed and measured using a variety of different physical principles [4]. The commonly used sensors to measure static moderate magnetic fields are based on the Hall effect, on a change in resistance caused by the magnetic field (anisotropic magnetoresistance (AMR) and giant magnetoresistnce (GMR)) or on the bistability used in fluxgate magnetometers. One possible route to new static magnetic field detection technologies is to use new physical phenomena [4].

In recent years, the effect of a time-varying magnetic field on systems with chaotic dynamics has attracted considerable attention [5,6,7,8,9]. Recently, Chua’s circuit [10] operating in a chaotic regime was used to experimentally present the stochastic resonance phenomenon for a weak periodic magnetic field signal [5], and the dynamic behavior of two Chua’s circuits coupled by the effect of mutual inductance has been reported [6]. The effect of the magnetic field on chaotic systems is quantified by different methods. The most commonly used method to quantify the effect of the magnetic field on a chaotic system relates variations in the measured magnetic field with deformations of a chaotic attractor. The metrics characterizing the attractor can be different geometric shape parameters. The changes in the geometric parameters of the attractor of a chaotic electronic circuit with an inductive element were used to detect the presence of a metallic target [7]. The dynamic behavior of a nonlinear system exhibits several different regimes and bifurcation points indicated by the bifurcation diagram. The regime change can be very fast, with an important change in the dynamic trajectory. If the operating point of the nonlinear system is adjusted near a switching threshold in the bifurcation diagram, any small change in a target signal generates a dramatic change in the system’s dynamic trajectory, which can easily be detected. Hu and Liu [8] used a Duffing oscillator operating on the verge of chaos and a large-scale periodic state to detect a weak sinusoidal signal from the detecting head of a metal detector. By the real-time calculation of Lyapunov exponents, they determined whether the Duffing oscillator was in the chaotic state or not. Recently, a proximity sensor to detect a metal target using a planar coil as a sensing element and the chaotic electronic circuit being the analog model of the Sprott Case N system [11,12] as the measurement unit was proposed [9]. The presence of a target was determined by the switch between periodic and chaotic oscillations in the circuit. The bandpass filter method was used to distinguish the oscillation mode. The effect of a static magnetic field on systems with chaotic dynamical behavior has not been experimentally studied so far.

Sensors based on chaotic systems and using their property that a small sensed signal change causes significant changes in system behavior have very high sensitivity in narrow ranges of the sensed signal. This possibly explains the very high sensitivity of natural sensing systems [13]. The attractor of a nonlinear system can consist of parts concentrated in different regions of the phase space, with fast transitions in the dynamic trajectory between these parts. A bistable sensor based on these chaotic dynamics requires neither an external driving signal (as in fluxgate magnetometers [14]) nor noise (as in noise-activated sensors [15]) for its functioning [16]. Its output can be quantified by the spike count rate [17], with spikes defined by times of dynamic trajectory crossings between attractors. The spike count rate is used to transmit the information about the stimulus in biological neurons [18]. A method to quantify the static magnetic field intensity by the spike count rate in systems with chaotic dynamical behavior has not been introduced so far.

The aim of this paper is to present experimental results obtained using an electronic Chua’s circuit operating in a chaotic regime where a double-scroll attractor exists [19,20], with the coil placed in a static uniform magnetic field. We quantified the dynamical behavior of the circuit by the duty cycle factor and the spike count rate, with spikes defined by crossing points between the scrolls of the double-scroll attractor. Our results were used to propose two sensors to detect and measure static and low-frequency moderate magnetic fields. They can have very high sensitivity resulting from the operation on the verge of different chaotic regimes and the output coded by the spike count rate used in coding neuronal responses to stimulus.

The paper is organized as follows: in Section 2, we present the experimental setup and describe the experimental procedure. Section 3 contains the experimental results and their discussion. In Section 4, the sensor for moderate static magnetic fields is proposed. Section 5 concludes the paper.

## 2. Materials and Methods

**Figure 1:**
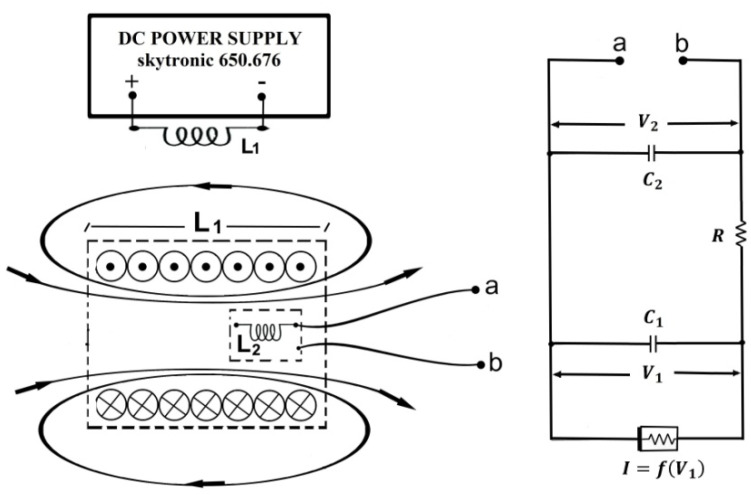
Chua’s circuit (**right plot**) with the inductor $L_{2}=18$ mH placed in the coil $L_{1}=85.1$ mH powered by DC (**left plot**), capacitors $C_{1}=10$ nF and $C_{2}=100$ nF, the nonlinear Chua diode with piecewise-linear characteristic $I=f\left(V_{1}\right)$ and variable resistor R.

Our experimental setup is shown in Figure 1 together with the parameters of the electronic components. Its main part is Chua’s electronic circuit [10] assembled according to instructions given by Kennedy [21] using two op amps TL084CN. Our Chua’s circuit is shown and described with details in the reference [22]. The 18 mH cylindrical inductor $L_{2}$, with a shield and core made of ferrite material (Bourns RL181S-183J-RC, Bourns, Riverside, CA, USA), was placed inside an air core large coil $L_{1}$ with inductance of 85.1 mH, a square cross-section of 3 cm at the side, a length of 6 cm and 1800 turns carrying an electric current from a DC power supply, the skytronic 650676. This coil produced a nearly uniform static magnetic field inside a region. We measured this magnetic field using a PHYWE teslameter (PHYWE, Göttingen, Germany) without Chua’s inductor inside.

**Figure 2:**
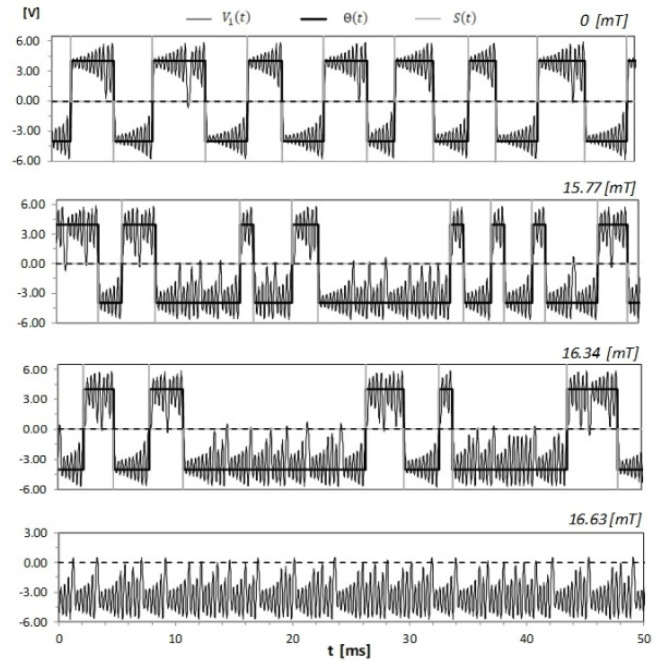
The temporal evolution of the voltage $V_{1}\left(t\right)$ with the step function Θ(t) identifying scrolls and its derivative - the spike function S(t) identifying crossing points between scrolls for R=1580 Ω and different static magnetic field intensities.

By reducing the variable resistor R, Chua’s circuit exhibits a sequence of bifurcations and it can be in equilibrium, periodic, period-doubling, single-scroll or double-scroll regimes [19,20]. The same sequence of bifurcations can be observed as Chua’s circuit inductor $L_{2}$ increases [20]. In our case, the bifurcation from the period-doubling to the single-scroll regime occurs for R decreased to 1687 Ω and from the single-scroll regime to the double-scroll regime for R decreased to 1645 Ω. Our experiments were performed in a chaotic double-scroll regime [19]. The structure of the chaotic strange attractor in this regime consists of two scrolls located in different regions of the phase space connected by swirling lines corresponding to fast jumps between them. Each scroll is characterized by irregular oscillations with frequencies around a certain dominant value, which, in our case, was 2.89 kHz. The time of a jump between scrolls is around the preferred rotation period, i.e., 0.35 ms. This time is shorter than the time of a spike emitted by a neuron [18], which is around 1–2 ms. The dynamic trajectory can easily be assigned to a given scroll by following the voltage $V_{1}\left(t\right)$ on the capacitor $C_{1}$. The voltage $V_{1}\left(t\right)$ oscillates around a positive or negative point, and, after a number of oscillations, it changes the point. We ascribed $V_{1}\left(t\right)$ to a given scroll after its passing through zero and at least two oscillations around its center. We recorded the voltage $V_{1}\left(t\right)$ for different static magnetic field intensities during a 10 s period, with a sampling rate of 50 kHz, using the oscilloscope Agilent DSO6032A. In Figure 2, we show examples of the temporal evolution of the voltage $V_{1}\left(t\right)$ for R=1580 Ω and different static magnetic field intensities. This figure was produced from stored data. One may notice that an increasing magnetic field intensity increases the residence time of the dynamic trajectory in one scroll and finally leads to a change in the chaotic attractor from double-scroll to single-scroll. This is due to the action of a static magnetic field on the core of Chua’s circuit inductor $L_{2}$. It moves the operating point in the saturation curve of the inductor core and allows one to control the voltage drop on the inductor. A static external magnetic field has an effect on the inductor only when its core saturates. When the core is saturated, its incremental permeability (µ) is greatly reduced; therefore, the inductance drops and the induced current disappears. The voltage applied to the circuit is opposed only by the voltage drop caused by the inductor winding resistance. The studied effect of a static magnetic field on Chua’s circuit can be modeled by introducing equations describing this circuit’s time-dependent incremental magnetic permeability and taking into account the saturation curve of the inductor core.

The temporal evolution of the voltage $V_{1}\left(t\right)$ with the step function Θ(t) identifying scrolls and its derivative—the spike function S(t) identifying crossing points between scrolls for R=1580 Ω and different static magnetic field intensities.

## 3. Results and Discussion

**Figure 3:**
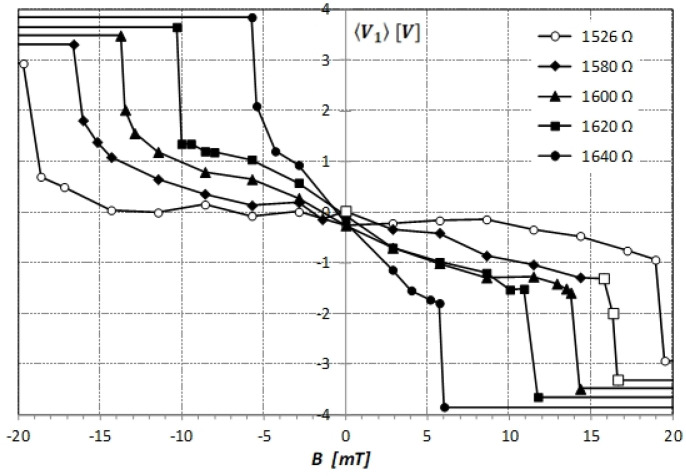
Experimental dependence of the average voltage $\langle V_{1}\left(t\right)\rangle$ on the capacitor $C_{1}$ calculated in 10 s time window on the intensity of the static magnetic field for different R values given in the plot. Examples of $V_{1}\left(t\right)$ for points denoted by empty squares are shown in Figure 2.

We recorded the voltage $V_{1}\left(t\right)$ time series on the capacitor $C_{1}$ during a 10 s period, with a sampling rate of 50 kHz in a chaotic double-scroll regime of Chua’s circuit for different values of the variable resistor R and different static magnetic field intensities B. Next, we calculated the time average $\langle V_{1}\left(t\right)\rangle$. This average is a duty cycle factor used to characterize continuous changes in the chaotic attractor with variations in the target signal [16]. The obtained results are presented in Figure 3. For moderately high values of the magnetic field intensity, $\langle V_{1}\left(t\right)\rangle$ increases or decreases slowly with B depending on its direction. The rate of this variation depends on the value of the resistivity R of Chua’s circuit and it is the highest for large R values. For a sufficiently high magnetic field intensity, the chaotic attractor changes to single-scroll in a very narrow range of B below 1 mT. One must proceed slowly to observe and record $V_{1}\left(t\right)$ in this transition region, which is extremely sensitive to the magnetic field intensity variations. The average $\langle V_{1}(t)\rangle$ changes by approximately two volts in this range, which can be detected.

**Figure 4:**
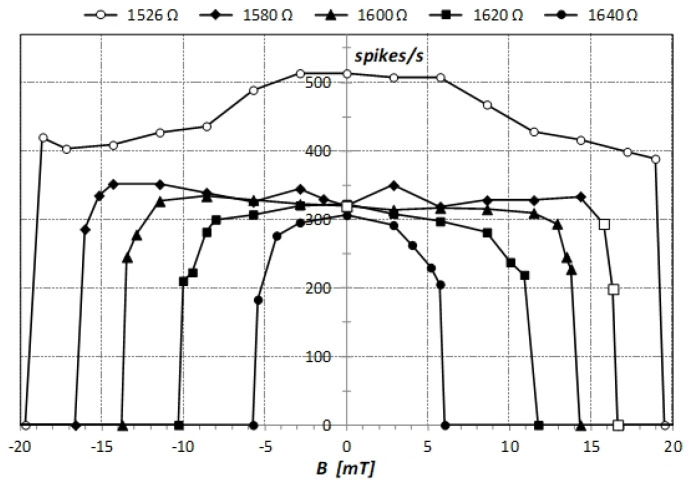
Experimental dependence of the spike count rate obtained in 10 s time window on the intensity of the static magnetic field for different R values given in the plot. Examples of $V_{1}\left(t\right)$ for points denoted by empty squares are shown in Figure 2.

The dynamic trajectory of a chaotic system with parts of the attractor located in different regions of the phase space can be quantified by a point process, with points defined by crossings between different parts. We followed the voltage $V_{1}\left(t\right)$ and ascribed each recorded sample to one of the two scrolls using the step function Θ(t). This function is a train of binary values with variable duration. Its derivative S(t) defines crossing points between scrolls and it can be identified with spikes of firing neurons. Examples of the voltage $V_{1}\left(t\right)$ together with the step function Θ(t) and the spike function S(t) are shown in Figure 2. In experiments, the concept of the spike count rate, defined as the number of spikes in the observation time window, is widely used [17,18]. In Figure 4, we present the obtained dependence of the spike count rate on the static magnetic field intensity for different values of the resistivity R of Chua’s circuit. For R>1530 Ω and a moderately high magnetic field intensity, the spike count rate does not depend on B. For smaller R values, the spike count rate increases with decreasing R and depends on B. It is the highest for B near zero and slowly decreases for higher ⎣B⎦ values. For a certain magnetic field intensity that depends on R, the chaotic attractor changes to single-scroll and spikes disappear. The fast and large drop in the spike count rate in a very narrow range of B indicates the high sensitivity of the system in this region of B values.

Our experiment shows that the choice of the observable used to characterize the effect of the static magnetic field on Chua’s circuit matters. At the threshold intensity of the static magnetic field, the average of the voltage $V_{1}\left(t\right)$ on the capacitor $C_{1}$ changes sharply by a few volts. This metric is easy to obtain, but it takes time to calculate it. The change in the spike count rate at the threshold is very large, between zero and around 320 spikes per second. Moreover, the appearance or disappearance of spikes at the threshold can be observed immediately. This metric is thus better for determining the threshold value.

## 4. Sensor Proposal

**Figure 5:**
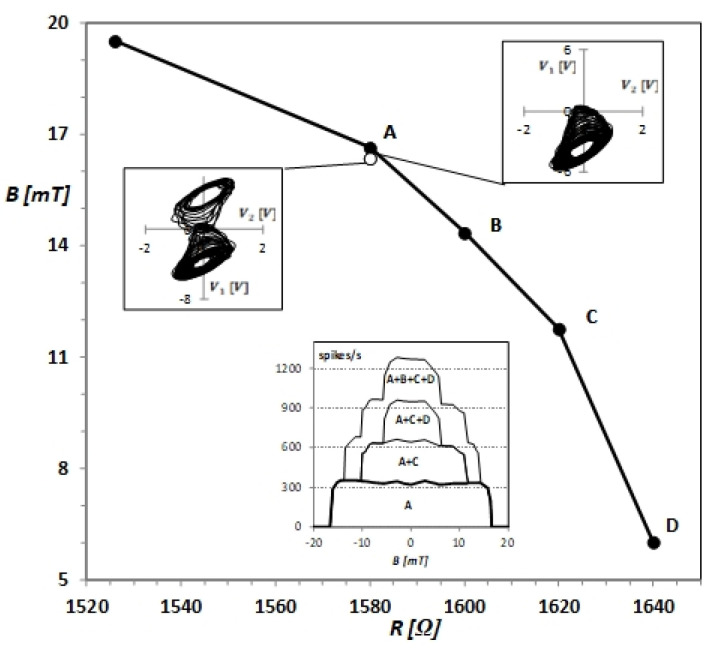
The experimental dependence of the static magnetic field intensity *B* on the resistivity *R* at the bifurcation threshold. The upper insets show phase portraits of Chua’s circuit corresponding to examples of $V_{1}\left(t\right)$ shown in Figure 2 for *B* equal to 16.34 mT and 16.63 mT. The lower inset presents transfer functions of sensors consisting of 1, 2, 3 and 4 Chua’s circuits with resistivities corresponding to points A–D and the output obtained by summing their spike count rates in 10 s time window.

Our experiments showed that, for each resistance R of Chua’s circuit, there are fast jumps between the two scrolls of the double-scroll attractor for static magnetic field intensities up to the very narrow region near the threshold, where the dynamic trajectory remains on one scroll and jumps disappear. The spike count rate falls from several hundred to zero in a range of B values below 1 mT. The Chua’s circuit is very sensitive to changes in B in this range. The experimental dependence of the threshold B value on R is presented in Figure 5. We show in this figure phase portraits of Chua’s circuit for R=1580 Ω and static magnetic field intensities 16.34 mT and 16.63 mT corresponding to examples of $V_{1}\left(t\right)$ time series given in Figure 2. One can observe that the threshold ⎣B⎦ value increases with decreasing resistivity R. These obtained experimental results have allowed us to propose two sensors to detect and measure moderate static magnetic fields.

The first sensor is based on one Chua’s circuit with a variable resistor R that operates in the chaotic double-scroll regime or in the single-scroll regime depending on the external static magnetic field intensity acting on the circuit inductor. In the double-scroll regime, the fast jumps between the two scrolls of the attractor generate jumps defined by crossing points between scrolls. In the single-scroll regime, there are no spikes. The transition between these two regimes is at the very narrow range of the static magnetic field intensity near the bifurcation. The static magnetic field between 5 and 20 mT can be measured by changing the resistance R and finding its value for which spikes appear or disappear. This value determines the bifurcation point between double-scroll and single-scroll chaotic attractors. The static magnetic field intensity is obtained from the dependence shown in Figure 5.

The second sensor consists of several Chua’s circuits with coils arranged at a distance that prevents their coupling and the mutual inductance effect [6]. Its output is defined by combinations of individual circuits’ spike count rates. In Figure 5, the lower inset presents the transfer functions of sensors consisting of one, two, three or four Chua’s circuits with resistivities of 1580 Ω (A), 1600 Ω (B), 1620 Ω (C) and 1640 Ω (D). Their output was obtained by summing their component Chua’s circuits’ spike count rates in a 10 s time window. The sensor with four or more Chua’s circuits can be used to measure a moderate static magnetic field. It can have an almost linear transfer function approximated by the step function (see, e.g., the transfer function A + B + C + D in Figure 5 for B > 0). In Figure 5, there is only one example of a possible transfer function of the proposed sensor. In practice, using the proposed method, one can build a sensor with any transfer function required in its application.

## 5. Conclusions

We have presented experimental results obtained for an electronic Chua’s circuit with an inductor placed in a static moderate uniform magnetic field and operating in a chaotic double-scroll regime. This is the first experiment showing the effect of a static magnetic field on an electronic circuit with chaotic dynamical behavior. This effect we quantified by two metrics: the duty cycle factor and the spike count rate, with spikes defined by crossing points between the scrolls of the double-scroll attractor. We observed a slow, monotonic, continuous variation in the duty cycle factor and a constant spike count rate for magnetic field intensities up to the narrow range below 1 mT, near the threshold at which the dynamic trajectory remains on one scroll and spikes disappear. In this range, both metrics change rapidly and severely, which can easily be noticed and detected. The threshold magnetic field intensity increases with the decreasing resistivity of the Chua’s circuit. The very high sensitivity of the Chua’s circuit near the threshold, which depends on the resistivity, allowed us to propose two sensors. The first sensor is based on one Chua’s circuit with a variable resistor and measures the static magnetic field intensity by determining the resistance value for which spikes appear or disappear. The second sensor consists of several independent Chua’s electronic circuits. Its transfer function is determined by the combination of individual Chua’s circuits’ spike count rates. This sensor can have a linear transfer function approximated by the step function, as in conventional sensors and sensors relating the slow continuous deformation of the chaotic attractor with variations in the measured signal. It can also have every desired transfer. Both sensors use dynamical sensing, i.e., very high sensitivity of a circuit to a small change in the magnetic field intensity when the dynamical regime changes. Finally, they convert a static moderate magnetic field into a form of spike rate, which parallels what happens in vivo in, e.g., nerve signaling.

## Data Availability

The data presented in this study are available on request from the corresponding author.
